# Is there an alternative treatment for patients intolerant to antiplatelet therapy if percutaneous left atrial appendage closure is considered?

**DOI:** 10.1007/s12471-017-0987-y

**Published:** 2017-04-20

**Authors:** F. Akca, N. J. Verberkmoes, S. E. Verstraeten, C. van Laar, B. P. van Putte, A. H. M. van Straten

**Affiliations:** 1 Department of Cardio-Thoracic Surgery, Catharina Hospital, Eindhoven, The Netherlands; 2 Department of Cardio-Thoracic Surgery, St. Antonius Hospital, Nieuwegein, The Netherlands

**Keywords:** Left atrial appendage, Left atrial appendage closure, Epicardial, Atrial fibrillation

## Abstract

**Introduction:**

Left atrial appendage (LAA) closure has become of major interest for patients with atrial fibrillation intolerant to oral anticoagulation therapy (OAC). Patients with a contraindication to both OAC and antiplatelet therapy are not eligible for percutaneous LAA closure. We aimed to find an alternative treatment for these specific patients.

**Methods:**

From March 2014 until December 2015 five patients were referred for percutaneous LAA closure. Alternative treatment was necessary due to an absolute contraindication to OAC and antiplatelet therapy (*n* = 4) or after previous failed percutaneous device implantation (*n* = 1). A stand-alone full thoracoscopic closure of the LAA using the Atriclip PRO device (AtriCure Inc., Dayton, OH, USA) was performed under guidance of transoesophageal echocardiography (TEE). After three months all patients underwent a computed tomography scan. Mean follow-up was 7.2 months [range 4.5–9.8 months].

**Results:**

All procedures were achieved without the occurrence of complications. Complete LAA closure was obtained in all patients without any residual flow confirmed by TEE. Postoperative computed tomography confirmed persisting adequate clip positioning with complete LAA closure and absence of intracardial thrombi. During follow-up no thromboembolic events occurred.

**Conclusion:**

For atrial fibrillation patients with an absolute contraindication to OAC and antiplatelet therapy a stand-alone, minimally invasive thoracoscopic closure of the LAA is a safe and feasible alternative treatment. This might be a solution to avoid serious bleeding complications while eliminating the thromboembolic risk originating from the LAA in patients who are not eligible for percutaneous LAA closure.

## Introduction

According to the current guidelines, oral anticoagulation (OAC) therapy is considered the first line treatment to reduce the risk of stroke in patients with atrial fibrillation (AF) with a CHA2DS2-VASc score ≥2 [1]. However, in some patients this lifelong OAC therapy results in excessive bleeding risks, even with the new OACs, as demonstrated in the RE-LY trial [2]. The PROTECT-AF trial demonstrated that occlusion of the LAA using a percutaneous closure device results in a stroke risk similar to that with OAC therapy [3, 4]. However, these percutaneous techniques require anticoagulation or antiplatelet therapy even after the implantation of the device [5, 6]. Furthermore, these approaches are associated with high rates of incomplete LAA closure varying from 13 to 32% during follow-up [7, 8]. For these patients, OAC therapy often cannot be discontinued and lifetime OAC use is warranted.

We aimed to find a solution to reduce the thromboembolic risk originating from the LAA in patients who are not eligible for percutaneous LAA closure. In this paper we present five patients with an absolute contraindication to OAC and antiplatelet therapy who were not eligible for percutaneous closure techniques, or had a previously failed percutaneous device implantation. We performed a stand-alone full thoracoscopic epicardial closure of the LAA using an epicardial clip device.

## Methods

### Patients

From March 2014 until December 2015 five patients were referred to the Catharina Hospital (Eindhoven) or the St. Antonius Hospital (Nieuwegein) to be discussed in the Electrophysiology Heart Team consisting of an electrophysiologist and a specialised cardiothoracic surgeon. The demographics are presented in Table 1. The patients presented in this paper were medically treated for AF and experienced either severe side effects on OAC/antiplatelet therapy or had an absolute contraindication. The patients were discussed to determine the best therapeutic strategy (conservative, medical therapy, percutaneous treatment or surgical intervention). In the patients described in this paper, a percutaneous LAA closure was considered infeasible or contraindicated and a stand-alone thoracoscopic closure of the LAA using an epicardial clip device (Atriclip PRO, AtriCure Inc., Dayton, OH, USA) was considered the most suitable alternative. According to the current guidelines, the included patients did not receive additional arrhythmia surgery, due to the absence of AF-related symptoms (European Heart Rhythm Association class I) [9]. Preoperative work-up included a chest X‑ray, transthoracic echocardiography to assess the left ventricular function, valvular function and presence of LAA thrombi, and standard laboratory tests.

**Table 1:** Demographics

Characteristic	Value
Age (years)	74 [70–83]
Gender (male)	4 (80%)
CHA2DS2-VASC score	5 [3–6]
HASBLED score	3 [2–5]
LVEF (%)	56 [47–59]
Previous PVI (*n*, %)	1 (20%)
Congestive heart failure/cardiomyopathy (*n*, %)	1 (20%)
Hypertension (*n*, %)	3 (60%)
Diabetes (*n*, %)	1 (20%)
Cerebrovascular accident (*n*, %)	3 (60%)
Vascular disease (*n*, %)	2 (40%)
Stabile coronary artery disease (*n*, %)	1 (20%)
Abnormal renal function (*n*, %)	2 (40%)
Prior bleeding (*n*, %)	5 (100%)
Pacemaker/ICD (*n*, %)	1 (20%)
COPD (*n*, %)	1 (20%)

Continuous variables are presented as median [range]

*COPD* chronic obstructive pulmonary disease, *ICD* implantable cardioverter defibrillator, *LVEF* left ventricular ejection fraction, *PVI* pulmonary vein isolation

#### Patient 1

**Fig. 1:**
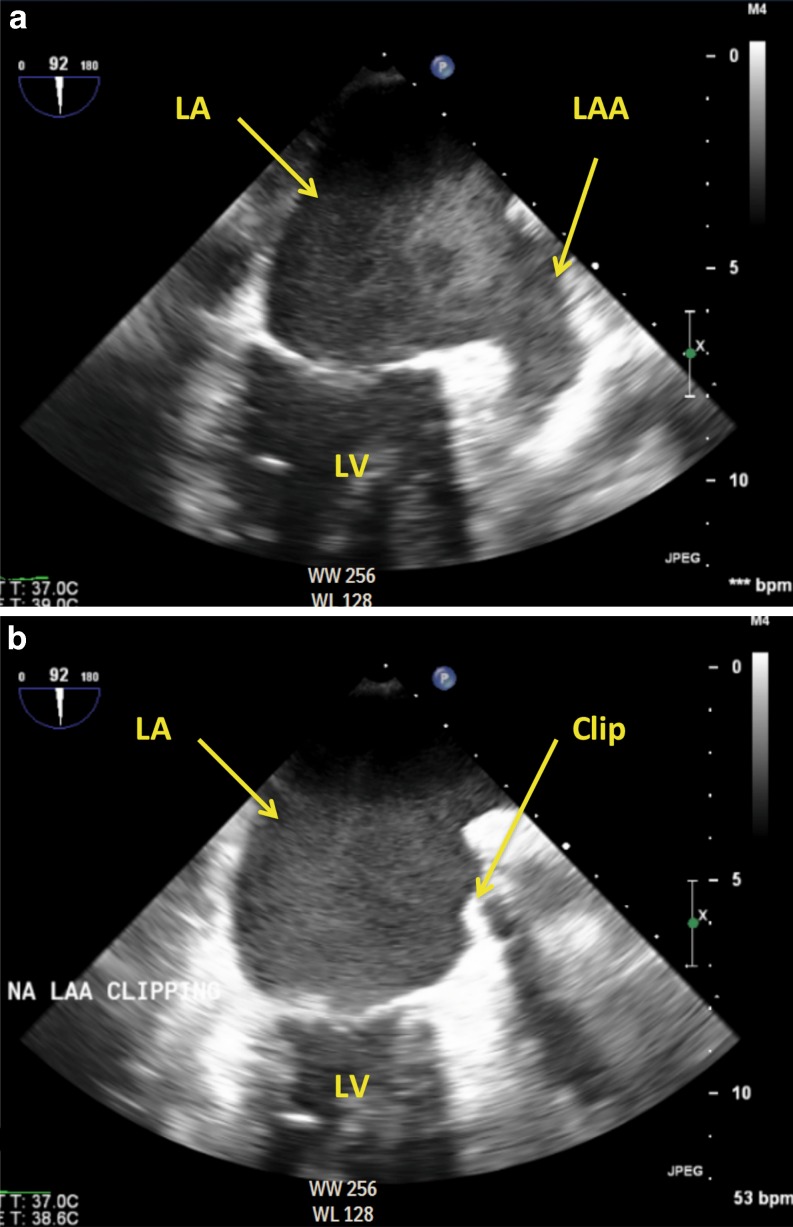
**a** Preoperative transoesophageal echocardiography demonstrating spontaneous contrast in the left atrial and left atrial appendage. **b** After placement of the Atriclip PRO device. Complete closure of the LAA is obtained. (*LA* left atrium, *LAA* left atrial appendage, *LV* left ventricle)

A 68-year-old male patient was referred with the request to evaluate the possibility for percutaneous LAA closure. The patient had a history of permanent, asymptomatic AF and Osler-Weber-Rendu disease. Despite a CHA2DS2-VASC score of 6, the patient was not treated with OAC or antiplatelet therapy and twice suffered a transient ischaemic attack. On echocardiography the patient had a giant left atrium with a diameter >7 cm with spontaneous echo contrast (Fig. 1). When treated with OAC or antiplatelet therapy he frequently experienced severe epistaxis resulting in a drop in the haemoglobin. In the workup for percutaneous LAA closure a test period of three weeks was introduced in which dual antiplatelet therapy (aspirin and clopidogrel) were prescribed to simulate the post-procedural treatment after percutaneous LAA closure. Within two weeks the patient suffered from severe epistaxis and the decision was made to perform LAA closure using the epicardial LAA Atriclip PRO device.

#### Patient 2

A 69-year-old female with AF was referred with a history of recurrent ischaemic stroke and recurrent intracranial bleeding due to multiple cavernous haemangiomas and cerebral amyloid angiopathy. Despite a CHA2DS2-VASC score of 5 the patient did not receive OAC because of her neurological status. The patient was referred for percutaneous LAA closure. After consultation with the treating neurologist it was determined that dual antiplatelet therapy was contraindicated due to the risk of intracranial bleeding and therefore percutaneous LAA closure was rejected.

#### Patient 3

A 74-year-old male was initially referred to our cardiology department to evaluate the indication for percutaneous LAA closure in the presence of a contraindication to OAC as therapy for AF. The patient had a history of bradycardia which required a VVI pacemaker, haemorrhagic stroke and ocular infarction. In spite of a CHA2DS2-VASC score of 3, OAC was contraindicated due to a previous haemorrhagic stroke. The patient was accepted for percutaneous LAA closure using the Watchman device (Boston Scientific, Marlborough, MA, USA). During the percutaneous procedure multiple attempts were performed for adequate placement of the device. However, due to recurrent dislocation this procedure was terminated. Subsequently, the patient was referred to the cardiothoracic surgery department for thoracoscopic epicardial closure of the LAA.

#### Patient 4

A 74-year-old male patient with AF and a history of a partial stomach resection presented with recurring gastrointestinal bleeding while on new OAC (dabigatran) therapy. Furthermore, he developed a peptic ulcer, which required the use of a proton pump inhibitor, and withdrawal of antiplatelet therapy to avoid life-threatening gastrointestinal bleeding. Since this patient could not use antiplatelet agents, percutaneous LAA closure was contraindicated.

#### Patient 5

An 83-year-old male presented with recurrent bleeding in the digestive tract while on both warfarin and new OAC therapy for AF. Furthermore, he had chronic anaemia which required frequent blood transfusion. After extensive investigation, OAC therapy was indicated as the primary cause by a gastroenterologist and haematologist and other pathology was excluded. To avoid additional gastrointestinal blood loss and to protect the intestinal wall, antiplatelet therapy was strongly discouraged.

### LAA closure procedure

All patients were placed in the supine position, underwent general anaesthesia and were intubated with a double lumen endotracheal tube. A transoesophageal echocardiography (TEE) probe was introduced and the LAA was visualised to ensure the absence of thrombi. For adequate pericardial vision, introduction of working instruments and device implantation, a standard left-sided minimally invasive thoracoscopic approach was used. The maximum incision length was 15 mm [range 5–15 mm]. The phrenic nerve was identified and a posterior pericardiotomy was performed to visualise the LAA. In all patients the Atriclip Pro LAA Exclusion System was used. The base of the LAA was measured intraoperatively and sized for the appropriate AtriClip Pro length (either 35, 40, 45 or 50 mm), which is independent from the anterior-posterior dimension of the left atrium as assessed by echocardiography. This system has been extensively described in previous papers [10–12]. The device consists of a self-closing clip manufactured of two parallel titanium tubes with elastic nitinol springs covered by braided polyester. The delivery system allows redeployment and repositioning ensuring optimal placement at the LAA base, resulting in a sealed line at the level of the former LAA orifice. After placement and before final release of the clip, the position of the device was confirmed by intrapericardial direct vision and by TEE. If a residual LAA flow or stump was observed the device was repositioned. After the procedure a chest tube was inserted in the left-sided costodiaphragmatic recess through one of the ports.

### Postoperative care

Patients were extubated in the operating room, observed on the post anaesthetic care unit and transferred to the ward on the same day. Oral anticoagulation and antiplatelet therapy were discontinued after the procedure.

### Follow-up

As part of our standard-of-care protocol, all patients were evaluated 6 weeks after discharge by a cardiothoracic surgeon specialised in cardiac electrophysiology. Three months after the procedure, patients underwent a CT scan to evaluate the position of the Atriclip PRO, any intracardiac thrombus and the presence of a residual ostium.

## Results

**Fig. 2:**
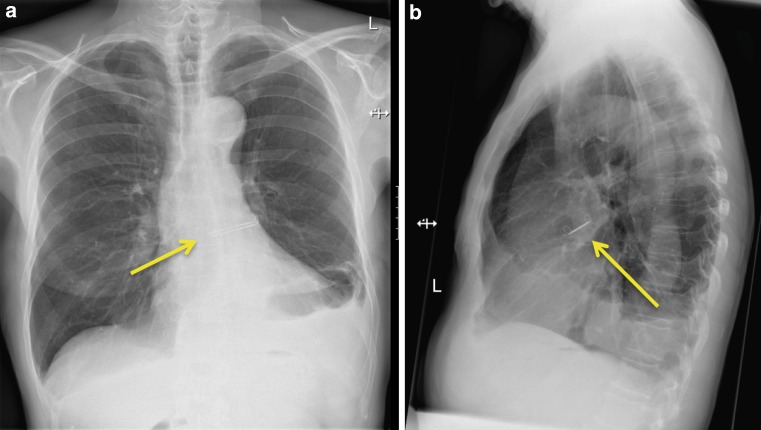
X-ray displaying the position of the Atriclip PRO device

All procedures were performed successfully without the occurrence of complications. The mean procedural duration was 52 min [range 33–59 min]. In one patient suboptimal placement of the device with respect to the LAA base was identified on TEE – resulting in a residual LAA ostium – and was successfully addressed after clip repositioning with adequate closure of the LAA during the same procedure. The clip size ranged from 40 to 50 mm. Clip positioning is demonstrated in Figs. 1 and 2. The postoperative period on the post anaesthetic care unit was uneventful and all patients were transferred to the ward on the same day. No complications occurred during hospital admission. Four patients (80%) were discharged uneventfully within a week. However, in the first patient a prolonged hospital stay occurred due to optimisation of antiarrhythmic drug therapy.

**Fig. 3:**
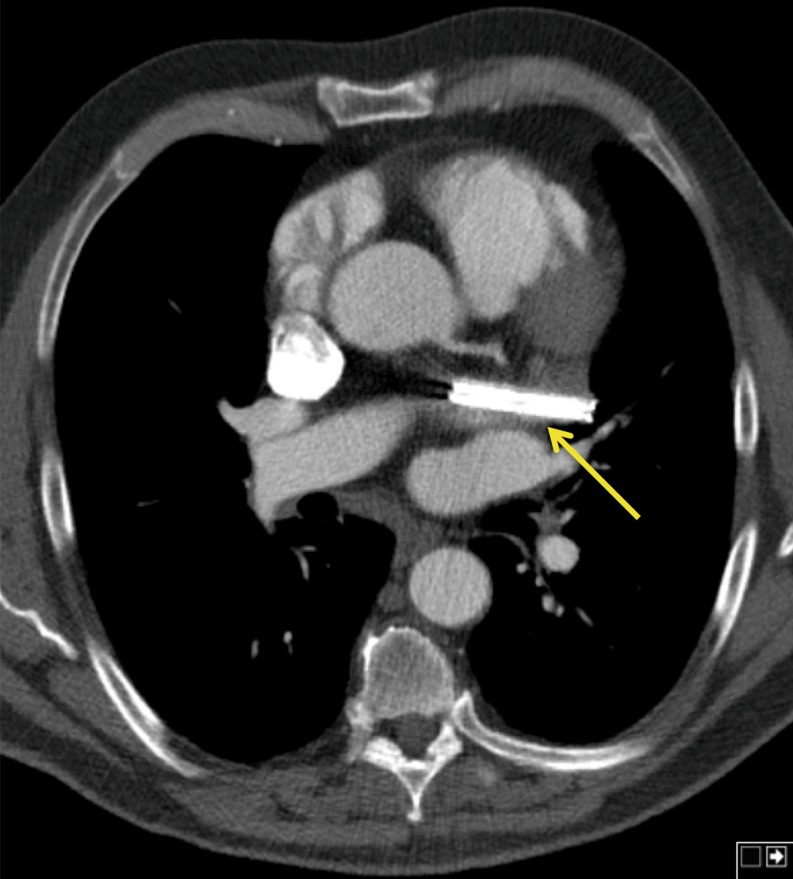
CT scan illustrating the position of the Atriclip PRO device. The clip is in adequate position with complete closure of the LAA

During a mean follow-up of 7.2 months [range 4.5–9.8 months] no patients experienced a stroke or transient ischaemic attack. In patient 1 the epistaxis recrudesced after discharge due to accidental use of aspirin. No recurrent episodes occurred after discontinuing the drug. Postoperative CT scans demonstrated adequate closure of the LAA without a residual neck >1 cm (Fig. 3), persistent adequate positioning of the Atriclip PRO device without dislocation, absence of intracardial thrombi or other anomalies.

## Discussion

The involvement of the LAA in AF-related thromboembolic stroke has been well established. Previous studies report that the LAA is the source of thrombi in up to 90% of the patients [13]. The PROTECT-AF trial, designed as an unblinded non-inferiority study, demonstrated that patients with an occluded LAA had comparable stroke rates to patients treated with OAC therapy [3, 4]. However, the percutaneous devices require OAC after implantation, temporary dual antiplatelet therapy and lifelong aspirin [5, 14]. We presented five cases of patients with AF who had absolute contraindications to OAC therapy in whom lifelong antiplatelet therapy was hazardous or after previous percutaneous treatment failure. In our series of patients a stand-alone full thoracoscopic approach for closure of the LAA using an epicardial clip device was effective and highly feasible. Procedures are minimally invasive and can be performed with short procedural times and fast recovery. None of our patients experienced any thromboembolic events during follow-up, despite their high risk as predicted by the CHA2DS2-VASc score.

Closure of the LAA is a well-established approach during arrhythmia surgery or with concomitant cardiac surgery [15, 16]. Surgical LAA removal or closure performed during concomitant arrhythmia or mitral valve surgery was demonstrated to be feasible and safe by the LAAOS trial [17]. With a long-term follow-up of 13 years Katz et al. demonstrated that remnant LAA flow is a risk factor for thromboembolic events [18]. Therefore, ligation and closure techniques resulting in incomplete LAA closure with any remnant flow are strongly warranted by these investigators. High rates of remnant flow are also observed in modern closure strategies ranging from 10 to 32%, such as in the Watchman (Boston Scientific, Marlborough, MA, USA) and Lariat (Sentre HEART Inc., Redwood City, CA, USA) devices [7, 8]. In various recent papers – in contradiction to earlier knowledge – it was stated that a remnant orifice with a high flow does not increase thromboembolic risk or even has a protective effect [7, 8]. From a historical point of view, we have major concerns about this new ‘remnant high flow’ theory. We believe that the available evidence on percutaneous devices is too limited – with scarce follow-up – to support such theory. Our opinion is that patients with an absolute contraindication to any anticoagulation strategy should undergo a treatment with the highest chance of complete closure of the LAA, in order to eliminate the need for lifelong OAC.

One of the advantages of thoracoscopic closure of the LAA using the Atriclip device is the ability to treat patients with anatomic variances and large LAA ostia. The LAA anatomy varies widely with an oval shaped ostium in the majority of patients (70%), but very different morphology is frequently found [19]. Percutaneous LAA closure using the Watchman device and the Amplatzer cardiac plug is limited by the maximum ostial diameter and therefore not feasible for some patients due to technical considerations and will potentially result in dislocation of the device [20]. The Atriclip PRO can be placed on the LAA under endoscopic vision of the cardiac surgeon irrespective of the LAA anatomy or left atrial dilatation and is not restricted by ostial size. The system can be deployed and placement can be adjusted at the discretion of the surgeon under guidance of TEE. Using the technique described in our paper, closure of the LAA can be performed on a beating heart and appropriate placement of the Atriclip can be secured without leaving a residual ostium. The endocardial closure lines at the base of the LAA consist of adjacent smooth natural endocardial tissue and in our patients we discontinued the use of OAC postoperatively without antiplatelet therapy [21].

From previous research it is known that removal of the atrial appendage results in changes in atrial natriuretic factor (ANF) levels. Recently, Maybrook and colleagues measured ANF levels after amputation of the LAA performed with the Lariat device [22]. Immediately after LAA closure there is a possible decrease in ANF levels leading to fluid retention followed by an excessive release of ANF with a significant natriuretic and diuretic effect 24–48 h post LAA closure. During the first weeks the ANF production from the LAA will decrease and will be compensated by increased production from the right atrium resulting in normal ANP levels. Care must be taken for patients with a pre-existing cardiomyopathy and the risk of fluid overload after LAA closure. Therefore, when closure of the LAA is indicated for these patients, appropriate titration of perioperative diuretics and antihypertensive agents must be considered.

We recognise that more research on epicardial LAA clipping is required; however, studies performed on the Atriclip system demonstrated that residual flow can be avoided without leaving a residual ostium >1 cm (which is a predictor for an increased thromboembolic risk) [11, 23]. During concomitant cardiac surgery closure of the LAA using the Atriclip device is currently a well-established technique and applied in many centres worldwide. Various other studies demonstrated that LAA occlusion using the Atriclip PRO device during thoracoscopic epicardial AF ablation is feasible and safe [24, 25]. As stand-alone treatment placement of the Atriclip PRO device is not well recognised with limited available evidence, which makes the use still off-label and in an experimental setting [10, 12]. To evaluate the best treatment strategy, we call for more international research to determine predictors and risk factors to assess whether patients have more benefit from either percutaneous or thoracoscopic LAA closure and whether it is safe to cease lifelong OAC and antiplatelet therapy.

In this paper we aimed to find a solution for AF patients in whom LAA closure is considered according to the guidelines and have 1) absolute contraindications to both OAC and antiplatelet therapy or 2) where failure of the closure device must be avoided due to the severity of contraindications or 3) where access issues prevent the possibility for implantation of percutaneous devices or 4) where other reasons result in a high probability of inappropriate percutaneous LAA closure and can lead to serious complications (e. g. in anatomic variances). We believe that stand-alone, thoracoscopic epicardial closure of the LAA using the Atriclip PRO device is a safe and feasible treatment and might be a solution to avoid serious bleeding complications while eliminating the thromboembolic risk originating from the LAA in patients who are not eligible for percutaneous LAA closure.
